# SOR Project: the French context

**DOI:** 10.1054/bjoc.2000.1756

**Published:** 2001-05

**Authors:** I Durand-Zaleski, T Philip

**Affiliations:** Department of Public Health, Henri Mondor hospital AP-HP, paris; Standards, Options et Recommandations FNCLCC, Paris; Centre Léon Bérard, Lyon, France


*British Journal of Cancer* (2001) **84** (Supplement 2), 4–5
© 2001 FNCLCC
doi: 10.1054/ bjoc.2001.1756, available online at http://www.idealibrary.com on IDEAL®

### SOR Project: the French context

**I Durand-Zaleski[1,2] and T Philip[2,3]**

[1]Department of Public Health, Henri Mondor hospital AP-HP, paris; [2]Standards, Options et Recommandations, FNCLCC, Paris; [3]Centre Léon Bérard, Lyon, France

Ever since the publication of the landmark article 'do practice guidelines guide practice?', clinicians and healthcare service researchers have attempted to address the issue of how best practice guidelines can guide practice (Lomas et al, 1989). Acceptance by clinicians of practice guidelines is at its best when guidelines are designed to improve patient outcome and not to reduce costs or to improve the indiscriminate endpoint known as 'quality of care' which is often considered as cost containment in disguise (Chassin, 1996). Cancer treatment appears to be favourable for the development of practice guidelines for at least two reasons; (a) variations in practice may be disadvantageous for patients (due in particular to differences in efficacy and side-effects of drugs) and b) evidence can be found that following recommended strategies results in improved patient survival, although there is the difficulty that statistical benefits are difficult to translate at an individual level.

### DEVELOPMENT OF GUIDELINES

Cancer treatment guidelines such as those established by the Standards, Options, Recommendations (SOR) possess some of the attributes that have been determined to be lead to good observance: they are evidence-based, worded in a non-ambiguous way and usually accompanied by decision trees (Grol et al, 1998). The SORs are written by a group of clinicians where all specialities concerned by a particular cancer are represented, as well as all types of institutions (public, teaching, private for-profit and not-for-profit hospitals, physicians in private practice). This point is important to ensure that, for example, surgeons will not have to follow recommendations crafted by radiotherapists. A survey of French clinicians found a reluctance for specialists to accept evidence from a group of other specialists. SOR do not require physicians involved in the treatment of cancer patients to acquire new skills and, as a rule, do not suggest that responsibility of management should be shifted from one type of physicians to another.

Cancer SORs have taken into account the most sensitive cultural aspects of the disease. As shown in the comparison between French and US guidelines on ovarian and breast cancer prevention, the criteria of medical evidence cannot help in areas of clinical uncertainties and is replaced by other criteria such as social preferences or values (Eisinger et al, 1999). Cultural adaptation of SORs is meant to ensure better compliance from physicians and acceptance by patients in matters where scientific information has not shown that a given strategy led to a better clinical outcome than another. It is achieved, via discussions with sociologists, clinical psychologists and patients' representatives.

### DISSEMINATION OF GUIDELINES

This does not mean, however, that guidelines for cancer treatment will be readily accepted by clinicians. As other authors have pointed out, acceptance depends not only on physicians' trust, but also on at least three other aspects: the magnitude of changes in practice imposed, and the removal of both administrative inefficiencies and negative financial incentives (Grol et al, 1998; Ellrodt et al, 1995). In the case of guidelines for cancer treatment, all three aspects are to be found to a certain extent depending on the topic. Cancer SORs require from physicians some changes in their practice and habits, but it has been shown that implementation of the standards was possible (Ray-Coquard et al, 1997).

The SOR patient project will allow the patient to participate in the implementation of standard procedures by knowing the best questions to discuss with his physician. Administrative inefficiencies may affect the management of cancer patients. One example is the recommendation of a multidisciplinary approach (Chardot et al, 1995; Dauplat et al, 1995; Bey et al, 1995; Heron et al, 1995). This multidisciplinary approach is possible only when all concerned parties are ready to collaborate and are given the means and the incentives to do so (Browman et al, 1995; Rodwin, 1993). The current organization and financing of the French healthcare system does not particularly promote multidisciplinary management of disease (despite repeated statements by the ministry that cancer treatments must be evidence-based and discussed among all specialists concerned), except perhaps in cancer centres where all specialities are to be found.

The promotion of a multidisciplinary approach requires that information networks are set up between professionals, with the funds to match and acknowledgement for physicians in private practice that discussion with colleagues amounts to a clinic visit and should be assigned a fee. In academic settings, where payment may be less of an issue, other non-monetary rewards may be used as reinforcers. Although we would like to believe that improving patient outcomes is enough of a reward, some body of evidence points to the opposite being true (Rodwin, 1993). Thus, it is important that participation in guideline development be acknowledged by academic institutions and appointment committees.

Negative financial incentives exist through the intricacies of the current organization of the French healthcare system. Public hospitals and not-for-profit cancer centres are funded via a capped yearly budget, which is not adjusted for the number or complexity of patients. Thus, any increase in the costs of chemotherapy has to be financed from the hospital's existing budget. Overspending is not permitted and results in carried-over debts, which will in turn lead to investment freeze and personnel reduction. Private for-profit hospitals have a different, fee-for-service reimbursement, with a capped fee for chemotherapy and an authorized mark-up over the purchase price, which may create an incentive to use expensive cytostatic drugs. Private hospitals benefit from full reimbursement for implantable devices, while public hospitals finance these on the same capped budget as drugs, personnel, etc. Thus, private for-profit centres have incentives to perform procedures and to use outpatient beds, while public and not-for-profit hospitals have an incentive to use whatever strategy has the lowest variable cost. In both the public and the private systems, financing interferes with the implementation of cancer SORs, although this is seldom addressed and has not been yet studied in a systematic way.

Another obstacle to the dissemination of guidelines is their number and the amount of paper they represent. The SORs published to date represent a pile close in height and weight to that described in the offices of general practitioners (Hibble et al, 1998), but they have the great advantage to be available in CD form and on the Internet. The computerized SOR with hypertext links solves the problem of easy access and timely retrieval of information, provided all physicians concerned are ready to use this medium. The advent of the Internet, while enabling some physicians to make better informed choices may also increase the gap between those who are familiar with this technology and those who are not. This may have several consequences, one of which is to shift informed patients from non-Internet doctors to Internet doctors, leaving together the most fragile populations of both doctors and patients.

### SPONSORSHIP AND CONFLICT OF INTERESTS

The SOR project is written by group of specialists coming mainly from the 20 French Cancer Centres, but also from university hospitals, general hospitals and private practice hospitals. All specialists are paid by their own employer and did not receive any salary (the average time for one guideline if a doctor participates in the writing group is 40 hours). Methodologists are systematically assigned to each group and at each stage of the process. Methodologists are full-time on the SOR project and they are paid by the Cancer Centres with government money. Elaboration of the various drafts are totally independent from industrial and pharmaceutical companies. However, the final draft dissemination is allowed to benefit from pharmaceutical companies' grants, but only for editing books or Internet materials. At this stage, pharmaceutical companies may not change anything from the approved text, which requires reviewing by a large panel of potential users before final approval by the group of experts and the FNCLCC.

### RELATIONSHIPS WITH GOVERNMENT AGENCIES

In France, ANDEM (National Agency for Medical Evaluation) had became ANAES (National Agency for Accreditation and Evaluation in the Healthcare System). ANDEM was an active participant in the SOR project initial phase, with specific help in methodology of guidelines and in various implementation techniques. However, the SOR project is independent from ANAES. The ANAES policy is moving more and more from guideline development to subcontract guidelines development leaving medical associations such as FNCLCC with control over the methodology.

The SOR programme is part of an international collaboration of researchers and policy makers who seek to improve the quality and effectiveness of clinical practice guidelines by establishing a shared framework for their development, reporting and assessment. The AGREE collaboration (Appraisal of Guidelines, Research and Evaluation) is an integrated research programme of the European Union and has the participation of a group of European countries: Denmark, Finland, France, Germany, Italy, the Netherlands, Spain, Switzerland and the United Kingdom, as well as Canada, New Zealand and the USA.

The authors are indebted to Martine Guesnier and to François Lemaire MD, for their insights into clinician's approach to quality assurance.

### REFERENCES

Bey P, Gaboriaud G, Peiffert D, Aletti P and Cosset JM (1995) Standards, options et recommandations pour une bonne pratique en radiothérapie externe et en curiethérapie en cancérologie. *Bull Cancer* **82**: 811–822

Browman GP, Levine MN, Mohide EA, Hayward RS, Pritchard KI, Gafni A and Laupacis A (1995) The practice guidelines development cycle: a conceptual tool for practice guidelines development and implementation. *J Clin Oncol* **13**: 502–512

Chardot C, Fervers B, Bey P, Abbatucci JS and Philip T (1995) Standards, options et recommandations pour une bonne pratique dans l'organisation pluridisciplinaire de la canceérologie. *Bull Cancer* **82**: 780–794

Chassin MR (1996) Quality of health care. Part 3: improving the quality of care. *N Engl J Med* **335**: 1060–1063

Dauplat J, Depadt G, Abbes M, Bobin JY, Carolus JM, Chardot C, Durand JC, Fondrinier E, Fraisse J, Guillemin F, Janser JC, Julien JP, Lallement M, Lasser P, Laurent JC, Lorimier G, Michel G, Pujol H, Rauch P, Rouanet P, Saint-Aubert B, Stockle E and Verhaeghe JL (1995) Standards, options et recommandations pour une bonne pratique en chirurgie cancérologique. *Bull Cancer* **82**: 795–810

Eisinger F, Geller G, Burke W and Holtzman NA (1999) Cultural basis for differences between US and French clinical recommendations for women at increased risk of breast and ovarian cancer [see comments]. *Lancet* **353**: 919–920

Ellrodt AG, Conner L, Riedinger M and Weingarten S (1995) Measuring and improving physician compliance with clinical practice guidelines. A controlled interventional trial [see comments]. *An Intern Med* **122**: 277–282

Grol R, Dalhuijsen J, Thomas S, Veld C, Rutten G and Mokkink H (1998) Attributes of clinical guidelines that influence use of guidelines in general practice: observational study. *BMJ* **317**: 858–861

Heron JF, Fervers B, Latour JF, Sommelet D, Chauvergne J and Philip T (1995) Standards, options et recommandations pour une bonne pratique en chimiothérapie. *Bull Cancer* **82**: 823–834

Hibble A, Kanka D, Pencheon D and Pooles F (1998) Guidelines in general practice: the new Tower of Babel? *BMJ* **317**: 862–863

Lomas J, Anderson GM, Domnick-Pierre K, Vayda E, Enkin MW and Hannah WJ (1989) Do practice guidelines guide practice? The effect of a consensus statement on the practice of physicians. *N Engl J Med* **321**: 1306–1311

Ray-Coquard I, Philip T, Lehmann M, Fervers B, Farsi F and Chauvin F (1997) Impact of a clinical guidelines program for breast and colon cancer in a French cancer center. *JAMA* **278**: 1591–1595

Rodwin M (1993) Medicine, money and morals. Oxford University Press: Oxford

© 2001 FNCLCC
